# Early detection of digestive system cancers: Insights from enzymatic and non-enzymatic tumour markers

**DOI:** 10.5937/jomb0-56390

**Published:** 2025-06-13

**Authors:** Thualfeqar Almohanna, Furqan Moein Auda, Basim M. Ali

**Affiliations:** 1 University of Kufa, Faculty of Medicine, Biochemistry Department, Iraq; 2 University of Kufa, Faculty of Science, Department of Chemistry, Iraq

**Keywords:** carcinoembryonic antigen, a-enolase, a-fetoprotein, calprotectin, colon cancer, rectum cancer, stomach cancer, digestive system cancer, karcinoembrionalni antigen, a-enolaza, a-fetoprotein, kalprotekcin, karcinom kolona, karcinom rektuma, karcinom želuca, karcinom digestivnog sistema

## Abstract

**Background:**

Digestive system cancer is a silent yet dangerous disease and represents a major cause of death worldwide. Among these cancers, colon cancer is the second leading cause of cancer-related deaths globally. This study aimed to detect malignant cells early by analyzing biochemical indicators associated with the transformation of digestive cancer cells. Tumour markers, primarily proteins associated with malignant cells, may play a role in preventing the spread and progression of the disease.

**Methods:**

In this study, the tumour markers analyzed included carcinoembryonic antigen (CEA), a-enolase (ENO1), a-fetoprotein (AFP), and calprotectin (CP) to detect digestive system cancer. These markers were measured in serum samples from sixty patients with digestive system cancer (the patient group) and sixty healthy individuals (the control group).

**Results:**

The results revealed highly significant changes (p-value <0.00001) in the levels of CEA (56.698±44.558 ng/mL), ENO1 (51.784±10.395 ng/mL), AFP (116.275±38.956 ng/mL), and CP (287.425±33.181 ng/mL) in the cancerous group compared to the control group, suggesting their potential utility in early diagnosis. Furthermore, significant differences (p-value <0.05) in liver enzymes and body mass index (BMI) provided additional evidence of pathological changes.

**Conclusions:**

The biomarkers investigated in this study demonstrate promising potential for early diagnosis while contributing to a deeper understanding of the biological mechanisms underlying digestive system cancers.

## Abstract (sr)

**Uvod:**

Karcinomi digestivnog sistema su tihi, ali opasni maligniteti i predstavljaju jedan od vodećih uzroka smrtnosti širom sveta. Među njima, karcinom kolona je drugi najčešći uzrok smrti povezanih s rakom na globalnom nivou. Cilj ove studije bio je rano otkrivanje malignih ćelija analizom biohemijskih indikatora povezanih sa transformacijom ćelija digestivnih karcinoma. Tumorski markeri, prvenstveno proteini povezani sa malignim ćelijama, mogu igrati ulogu u sprečavanju širenja i progresije bolesti.

**Metode:**

U ovoj studiji analizirani su tumorski markeri: karcinoembrionalni antigen (CEA), a-enolaza (ENO1), a-fetoprotein (AFP) i kalprotekcin (CP) za detekciju karcinoma digestivnog sistema. Ovi markeri su mereni u serumskim uzorcima dobijenim od šezdeset pacijenata sa karcinomom digestivnog sistema (grupa pacijenata) i šezdeset zdravih ispitanika (kontrolna grupa).

**Rezultati:**

Rezultati su pokazali statistički značajne promene (p-vrednost <0,00001) u nivoima CEA (56,698±44,558 ng/mL), ENO1 (51,784±10,395 ng/mL), AFP (116,275±38,956 ng/mL) i CP (287,425±33,181 ng/mL) kod pacijenata sa karcinomom u poređenju sa kontrolnom grupom, što ukazuje na njihov potencijal za ranu dijagnostiku. Pored toga, značajne razlike (p-vrednost <0,05) u nivoima jetrenih enzima i indeksu telesne mase (BMI) pružile su dodatne dokaze o patološkim promenama.

**Zaključak:**

Biomarkeri ispitani u ovoj studiji pokazuju obećavajući potencijal za ranu dijagnostiku, doprinoseći boljem razumevanju bioloških mehanizama koji leže u osnovi karcinoma digestivnog sistema.

## Introduction

Digestive system cancer is one of the most silent and deadly diseases, considered the second leading cause of cancer-related deaths worldwide [1]. Recently, many studies have shown a significant increase in colon cancer among individuals under the age of fifty [2] [3], potentially due to unhealthy lifestyle factors such as smoking, obesity, physical inactivity, and poor dietary habits [3]. Among the important nutrients in the diet, polyphenols can alter the composition and functionality of the microbiota, exerting a beneficial modulating effect on inflammation and free radicals [4]. These compounds may prevent and/or treat cancer by inhibiting critical pathways associated with colorectal cancer (CRC) [5]. Thus, it is imperative to discover the underlying interplaying factors responsible for the polyphenols-microbiota-CRC relationship to delineate a particular focus in formulating nutritional and therapeutic strategies [6] [7]. Tumour markers detect the earliest possible transformation of malignant cells [1]. These markers, often proteins associated with malignancies, can be clinically valuable in diagnosing and managing cancer patients [8]. Various classical markers have been used to identify colorectal cancers [9], including carcinoembryonic antigen (CEA), α-enolase (ENO1), α-fetoprotein (AFP) [9] [10], and calprotectin (CP) [11]. Carcinoembryonic antigen (CEA) is a glycoprotein discovered in 1965 by Gold and Freedman [12] [13]. Its levels rise in most patients with colon cancer, making it a potential early indicator for the detection of this type of cancer. However, it is important to note that this glycoprotein may also rise in infections affecting the liver, pancreas, and intestines [14].

The glycolytic enzyme α-enolase is overexpressed in several cancers and plays a role in the proliferation and metastasis of tumour cells. Cancer cells undergo characteristic changes in glucose metabolism to support their unrestricted proliferation and metastasis [10]. Enolase 1 (ENO1) is a conserved glycolytic enzyme that catalyzes the formation of phosphoenolpyruvate from 2-phosphoglycerate, an ATP-generating step that supports cancer cell proliferation and metastasis [10] [15]. In addition, ENO1 is involved in several physiological processes, such as cell growth, hypoxia tolerance, and autoimmunity [16]. Previous studies have reported ENO1 overexpression in several cancers, including breast [16] [17], neck, lung [18], prostate [19], and gastric cancer, where it is closely linked with cancer progression and poor patient prognosis [20]. Calprotectin (CP) is one of the most abundant proteins in human neutrophilic granulocytes and macrophages. In humans, it acts as an indicator of inflammatory and bowel diseases [21]. CP is a calcium-binding protein with functions analogous to those of inflammatory cytokines. Its levels increase in response to cancer, and scientists hypothesize that calprotectin (CP) influences intracellular calcium dynamics, thereby affecting processes such as cell cycle progression, survival, proliferation, differentiation, and migration [22].

## Materials and methods

Samples were collected from digestive system cancer patients after obtaining informed consent. Sixty samples were collected from patients with digestive system cancer (30 men and 30 women) at the Oncology Center in Najaf Governorate, including 20 cases of colon cancer, 20 cases of rectum cancer, and 20 cases of stomach cancer; their ages ranged from thirty to sixty years. Sixty samples were collected from apparently healthy individuals (control group). The study aimed to measure carcinoembryonic antigen (CEA), α-enolase (ENO1), α-fetoprotein (AFP), and calprotectin (CP) in serum samples as early indicators for detecting digestive system cancers, along with liver enzymes and body mass index (BMI). ELISA technology [23] [24] was used to measure carcinoembryonic antigen (CEA; Melsin’s MTM-0005 kit), α-enolase (ENO1; MyBiosource’s MBS706020 kit), α-fetoprotein (AFP; Melsin’s MTM-0006 kit), and calprotectin (CP; Melsin’s EKCAN-0005 kit). Liver enzymes were measured using spectrophotometry [25] [26]. Statistical analysis was performed using SPSS software (IBM SPSS Statistics, version 29.0.2.0, 2023) and Microsoft Excel (version 16.89.1, 2024), where the p-value, mean, and standard deviation were calculated [27]. Additionally, this study was approved by the University of Kufa and the Oncology Center in Najaf Governorate. The research adhered to relevant international and national guidelines for studies involving human participants. Written informed consent was obtained from all participants prior to their inclusion in the study.

## Results and discussion

The results presented in Table 1 demonstrate consistently elevated levels of CP and ENO1 in cancer patients alongside traditional tumour markers (AFP and CEA). Although variations in liver enzymes (ALP and ALT) were observed, these changes were less pronounced than the tumour markers. Additionally, while BMI showed statistical significance, its variation was minimal; hence, it may require further consideration regarding clinical interpretation. Notably, calprotectin showed a highly significant p-value (<0.00001), suggesting its role in the inflammatory mechanisms associated with cancer. Similarly, α-Enolase was statistically significant with a p-value of less than 0.00001, highlighting its role in tumour metabolic pathways. These results agree with other published studies [11] [28] [29], further reinforcing the relevance of these biomarkers in cancer pathology.

**Table 1:** Enzymatic and non-enzymatic tumour markers in digestive system cancer patients and control groups.

Parameters	Patients (n=60)	Healthy controls (n=60)	P-value
Calprotectin (ng/mL)	287.425±33.181	52.000±16.030	<0.00001
a-enolase (ng/mL)	51.784±10.395	6.848±2.280	<0.00001
a-fetoprotein (ng/mL)	116.275±38.956	3.179±1.435	<0.00001
Carcinoembryonic antigen (ng/mL)	56.698±44.558	1.146±0.862	<0.00001
ALP (U/L)	110.933±43.007	95.050±23.660	0.014
ALT (U/L)	20.250±7.308	17.200±7.695	0.028
BMI (kg/m^2^)	24.330±0.0600	25.561±2.671	0.006

The highly significant p-value for a-fetoprotein (AFP) (<0.00001) suggests that AFP could serve as a potential diagnostic tool for patients with gastrointestinal malignancies. Similarly, the significant p-value for carcinoembryonic antigen (CEA) (<0.00001) indicates considerable variability in CEA levels across the patient population. This variability may hold diagnostic utility in detecting diseases, consistent with findings from prior studies [29] [30] [31]. Liver Enzymes and BMI were also significant influences on the results of digestive system cancer. Alkaline phosphatase (ALP) levels significantly differed between patients and controls (p-value=0.014), indicating potential liver involvement. Alanine transaminase (ALT) levels were elevated in the patient population compared to controls, with a p-value of 0.028, which may suggest liver involvement or systemic effects of cancer. Body mass index (BMI) was slightly lower in patients compared to the healthy control group, with a p-value of 0.006. Although the difference was minimal, it may reflect metabolic or nutritional alterations in cancer patients. These findings are in agreement with previous studies [32] [33]. Additionally, Table 2 summarizes the demographic characteristics of the study participants, including age, sex, body mass index (BMI), and cancer type.

**Table 2:** Demographic characteristics of study participants.

Group	Age (years)	Sex (M/F)	BMI (kg/m^2^)	Cancer type
Digestive Cancer	30–60	30/30	24.330±0.060	Colon, Rectum, Stomach
Healthy Controls	28–63	30/30	25.561±2.671	N/A

**Figure 1:**
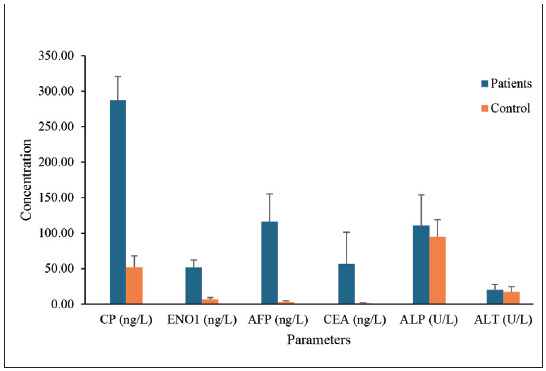
Comparison of enzymatic and non-enzymatic tumour marker levels in digestive system cancer patients and the control group.

**Figure 2:**
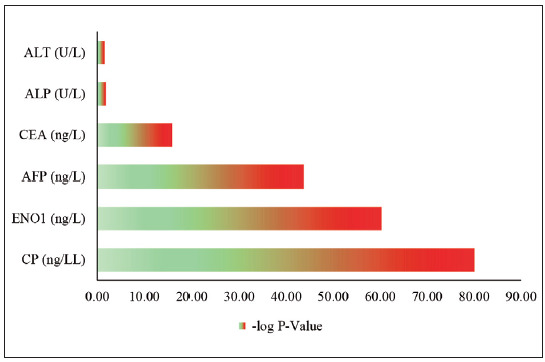
Analysis of the key tumour markers in digestive system cancer patients, with the t-test applied to calculate the p-value.

These findings strongly support the extremely low p-values observed for CP, ENO1, AFP, and CEA. All these biomarkers demonstrated a clear separation between cancer patients and healthy controls, further establishing their diagnostic potential, as shown in Figure 1. Therefore, the current results indicate that the α-enolase enzyme plays a significant role in the development and progression of gastrointestinal cancer. These findings are consistent with the study by Cheng et al. [10], which demonstrated a significant increase in α-enolase levels in colorectal cancer tissues compared to adjacent normal tissues. These results indicate that CP and ENO1 might be novel biomarkers for digestive system cancers. Their marked elevation in patients is consistent with their respective roles in inflammation and metabolic reprogramming [34] [35]. Combining these markers with traditional tumour markers, including AFP and CEA, may improve diagnostic accuracy and further elucidate the disease state. These associations of CP and ENO1 with traditional tumour markers suggest that these new markers could supplement the existing armamentarium of diagnostic tools. Thus, the marked differences in biomarker levels between cancer patients and healthy controls support the clinical utility of CP and ENO1 as non-invasive diagnostic markers. Their elevation in digestive system cancers aligns with their roles in inflammation and metabolic reprogramming, processes integral to cancer biology [10] [11] [36]. In addition, Figure 2 demonstrates the diagnostic and prognostic potentials of these markers regarding digestive system malignancies. The correlation of high levels of CP and ENO1 with AFP and CEA indicates their potential as part of a biomarker panel for early detection and monitoring of digestive system cancers. Though less pronounced, the significant differences in liver enzymes (ALP and ALT) between patients and controls may indicate liver involvement or systemic effects of cancer [32] [33].

This study presents the differential expression of biomarkers at significant levels, including calprotectin, α-enolase, carcinoembryonic antigen (CEA), and α-fetoprotein (AFP), in patients with various cancers of the digestive system, colon cancer, rectum cancer, and stomach cancer compared to normal controls. The high expression of CP and ENO1 in gastrointestinal cancers underscores their potential role as markers of inflammation and tumour progression [10] [21] [22]. Calprotectin, a known biomarker of inflammation, is released by activated neutrophils and macrophages. Its high level in the cohort of gastrointestinal cancer patients may indicate an association with the heightened inflammatory milieu linked to the process of tumourigenesis and cancer progression. Chronic inflammation in cancer, especially gastrointestinal malignancies, is a defined hallmark where prolonged inflammatory signalling contributes to the initiation, growth, and metastasis of tumours [11] [37].

Similarly, ENO1 is a multifunctional protein involved in a crucial step of glycolysis and cellular metabolism. Besides its metabolic role, ENO1 participates in multiple aspects of cancer development, such as angiogenesis and immune escape [38]. High levels of ENO1 in cancer patients may indicate metabolic reprogramming, a hallmark of cancer cells known as the ‘Warburg effect,’ where cancer cells preferentially utilize glycolysis for energy production even in the presence of oxygen [39] [40]. This shift in metabolism supports rapid proliferation and survival under a hypoxic tumour microenvironment [16]. In addition, ENO1 is also involved in critical cellular functions and has a significant role in tumour initiation and regression [39]. Several studies have demonstrated that ENO1 is a diagnostic marker in various tumours, and its overexpression positively correlates with cancer progression and poor prognosis [39] [41]. However, the role of ENO1 in colorectal cancer (CRC) remains poorly understood.

A previous study revealed that ENO1 is overexpressed in CRC tissues compared to normal colon tissues and promotes CRC cell proliferation and migration, suggesting that ENO1 plays an oncogenic role in CRC. This hypothesis was further supported by the evidence that ENO1 modulated the mTOR pathway in CRC cells [42]. Another study reported that Rab1A promotes CRC genesis and metastasis by targeting the mTOR pathway [43]. However, a potential molecular crosstalk between ENO1 and Rab1A in CRC cells remains unclear and needs to be elucidated. In addition, several studies have demonstrated a strong association between calprotectin (CP) and cancers of the soft tissues and digestive system [22] [43]. CP levels, along with inflammatory cells, were found to be significantly elevated in malignant tumours. Notably, fibrosarcomas exhibited a pronounced increase in inflammatory cells. However, some other malignant tumours showed lower levels of inflammatory cells despite elevated CP levels.

These findings also extend to the inflammatory conditions associated with digestive system cancers. It is well-established that chronic inflammation, driven by chemical and physical factors, significantly increases the risk of cancer development [22] [36]. Incorporating these biomarkers into routine diagnostic workflows could improve early detection, particularly with traditional markers like AFP and CEA. These biomarkers are established indicators of the presence and burden of a tumour, especially in gastrointestinal cancers. Further research is required to confirm the diagnostic and prognostic value of CP and ENO1 in more extensive, multi-centre cohorts. Longitudinal studies should also investigate their utility in monitoring treatment response and disease progression.

Additionally, mechanistic studies are necessary to elucidate the precise roles of CP and ENO1 in cancer biology, which may lead to identifying novel therapeutic targets. Furthermore, integrating CRISPR-Cas13 technology could significantly advance early prediction and detection capabilities [44]. Thus, these biomarkers represent some of the most significant tumour markers utilized in diagnosing digestive system cancers, each contributing critically to cancer detection and monitoring.

## Conclusion

The present study highlights the potential of ENO1 and CP as effective biomarkers for digestive system cancers. Elevated levels of these markers in cancer patients, along with their correlation with conventional (CEA and AFP) tumour markers, underscore their relevance in early cancer diagnosis and prognosis. This study opens up the prospects for further research toward developing a comprehensive diagnostic approach involving new (CP and ENO1) andtraditional (CEA and AFP) biomarkers for gastrointestinal malignancies. Overall, elevated levels of CEA confirm its utility as a marker for colorectal and gastrointestinal malignancies. High levels of CP are associated with inflammation, supporting its use as a non-invasive marker for gastrointestinal cancer, given its strong concordance with inflammatory processes. Moreover, the overexpression of ENO1 indicates its involvement in glycolysis and proliferation of cancerous cells, corresponding to the Warburg effect. These findings encourage using ENO1 as a non-invasivemarker for gastrointestinal cancers. Although changes in liver enzymes and BMI are less pronounced, they also signal the systemic effects of malignancy.

## Dodatak

### Conflict of interest statement

All the authors declare that they have no conflict of interest in this work.

## References

[1] Jelski W, Mroczko B (2020). Biochemical Markers of Colorectal Cancer: Present and Future. Cancer Manag Res.

[2] Cueva C, Gil-Sánchez I, Ayuda-Durán B, González-Manzano S, González-Paramás A, Santos-Buelga C, Bartolomé B, Moreno-Arribas M (2017). An Integrated View of the Effects of Wine Polyphenols and Their Relevant Metabolites on Gut and Host Health. Molecules.

[3] Harriss D J, Atkinson G, George K, Tim C N, Reilly T, Haboubi N, Zwahlen M, Egger M, Renehan A G (2009). Lifestyle factors and colorectal cancer risk (1): Systematic review and meta-analysis of associations with body mass index. Colorectal Dis.

[4] Espín J C, González-Sarrías A, Tomás-Barberán F A (2017). The gut microbiota: A key factor in the therapeutic effects of (poly)phenols. Biochem Pharmacol.

[5] Risan T Z, Ali B M (2024). AIP Conference Proceedings, AIP Conference, 2024.

[6] Mileo A M, Nisticò P, Miccadei S (2019). Polyphenols: Immunomodulatory and Therapeutic Implication in Colorectal Cancer. Front Immunol.

[7] Borzì A M, Biondi A, Basile F, Luca S, Vicari E S D, Vacante M (2018). Olive Oil Effects on Colorectal Cancer. Nutrients.

[8] Ali Numan H, Almohanna T, Almayali M Q, Adday Ali R, Khazal Abdulghareeb Taha H, Addai Ali H (2024). The effect of Clusterin level as a potential marker in women with polycystic ovary syndrome. Edelweiss Applied Science and Technology.

[9] Jue W, Lulu L, Yan Z, Gu S (2024). Nivoi ekspresije i dijagnostička vrednost serumskog GDNF, CEA i CA199 kod pacijenata sa kolorektalnim karcinomom. J Med Biochem.

[10] Cheng Z, Shao X, Xu M, Zhou C, Wang J (2019). ENO1 Acts as a Prognostic Biomarker Candidate and Promotes Tumor Growth and Migration Ability Through the Regulation of Rab1A in Colorectal Cancer. Cancer Manag Res.

[11] Jukic A, Bakiri L, Wagner E F, Tilg H, Adolph T E (2021). Calprotectin: From biomarker to biological function. Gut.

[12] Quentmeier A, Möller P, Schwarz V, Abel U, Schlag P (1987). Carcinoembryonic antigen, CA 19-9, and CA 125 in normal and carcinomatous human colorectal tissue. Cancer.

[13] Gold P, Freedman S O (1965). Specific carcinoembryonic antigens of the human digestive system. J Exp Med.

[14] Koness R (1995). CEA: Is it of value in colorectal cancer?. Rhode Island Medicine.

[15] Zhu X, Miao X, Wu Y, Li C, Guo Y, Liu Y, Chen Y, Lu X, Wang Y, He S (2015). ENO1 promotes tumor proliferation and cell adhesion mediated drug resistance (CAM-DR) in Non-Hodgkin's Lymphomas. Exp Cell Res.

[16] Gao J, Zhao R, Xue Y, Niu Z, Cui K, Fachang Y U, Zhang B O, Sheng L I (2013). Role of enolase-1 in response to hypoxia in breast cancer: Exploring the mechanisms of action. Oncol Rep.

[17] Tu S, Chang C, Chen C, Tam K, Wang Y, Lee C, Lin H, Cheng T, Huang C, Chu J, Shih N, Chen L, Leu S, Ho Y, Wu C (2010). Increased expression of enolase a in human breast cancer confers tamoxifen resistance in human breast cancer cells. Breast Cancer Res Treat.

[18] Tsai S, Chien I, Shen W, Kuo Y, Jin Y, Wong T, Hsiao J, Wang H, Shih N, Wu L (2010). ENO1, a potential prognostic head and neck cancer marker, promotes transformation partly via chemokine CCL20 induction. Eur J Cancer.

[19] Duijvesz D, Burnum-Johnson K E, Gritsenko M A, Hoogland A, Vredenbregt-van den Berg M S, Willemsen R, Luider T, Paša-Tolić L, Jenster G (2013). Proteomic Profiling of Exosomes Leads to the Identification of Novel Biomarkers for Prostate Cancer. PLoS One.

[20] Bai Z, Ye Y, Liang B, Xu F, Zhang H, Zhang Y, Peng J, Shen D, Cui Z, Zhang Z, Wang S (2011). Proteomics-based identification of a group of apoptosis-related proteins and biomarkers in gastric cancer. Int J Oncol.

[21] Stří' I, Trebichavský I (2004). Calprotectin-a pleiotropic molecule in acute and chronic inflammation. Physiol Res.

[22] Savaş O, İpek V (2021). Investigation of Calprotectin Positive Leukocytes in Canine Soft Tissue Tumors. Journal of Research in Veterinary Medicine.

[23] Shiojima Y, Moriyama H, Takahashi M, Takahashi R, Maruyama K, Bagchi M, Bagchi D (2022). Novel ELISA technology in assessing undenatured type II collagen in functional foods and dietary supplements used for knee joint health care: its sensitivity, precision, and accuracy. Functional Foods in Health and Disease.

[24] Almohanna T, Ahmed S A, Hussain M K (2012). Relevance of sex hormones levels with spermogram of infertile men. Global J Med Res.

[25] Rifai N (2023). Fundamentals of Clinical Chemistry and Molecular Diagnostics-E-Book.

[26] Auda F M, Ali A M, Dhyaa S (2021). Levels of Heavy Metal and Trace Element Among Children with Autism Spectrum Disorders. J Phys Conf Ser.

[27] Rathore G S (2023). Biostatistics and Research Methodology.

[28] Hsiao K, Shih N, Fang H, Huang T, Kuo C, Chu P, Hung Y, Chou S, Yang Y, Chang G, Liu K (2013). Surface a-Enolase Promotes Extracellular Matrix Degradation and Tumor Metastasis and Represents a New Therapeutic Target. PLoS One.

[29] Johnson P J (2001). The Role of Serum Alpha-Fetoprotein estimation in the Diagnosis and Management of Hepatocellular Carcinoma. Clin Liver Dis.

[30] Goldstein M, Mitchell E P (2005). Carcinoembryonic Antigen in the Staging and Follow-up of Patients with Colorectal Cancer. Cancer Invest.

[31] Duffy M J (2001). Carcinoembryonic Antigen as a Marker for Colorectal Cancer: Is It Clinically Useful?. Clin Chem.

[32] Dooley J S, Lok A S, Garcia-Tsao G, Pinzani M (2018). Sherlock's Diseases of the Liver and Biliary System.

[33] Giannini E G, Testa R, Savarino V (2005). Liver enzyme alteration: A guide for clinicians. CMAJ.

[34] Ali B, Mohammed U J (2024). The association of atherosclerosis with cortisol and Alpha-enolase levels and lipid profile. Anaesthesia, Pain & Intensive Care.

[35] Auda F M, Rehman A (2025). Evaluation of midkine, cortisol, and other biochemical indicators in patients with atherosclerosis. Anaesthesia, Pain & Intensive Care.

[36] Shokrgozar M A, Zali H, Rezaei-Tavirani M, Moghadamnia S (2007). Evaluation of proliferation inhibition effect of human calprotectin on human gastric cancer cell line (AGS) in vitro. Cell Journal (Yakhteh).

[37] Ross F A, Park J H, Mansouri D, Combet E, Horgan P G, Mcmillan D C, Roxburgh C S D (2022). The role of faecal calprotectin in diagnosis and staging of colorectal neoplasia: A systematic review and meta-analysis. BMC Gastroenterol.

[38] Díaz-Ramos À, Roig-Borrellas A, García-Melero A, López-Alemany R (2012). a-Enolase, a Multifunctional Protein: Its Role on Pathophysiological Situations. J Biomed Biotechnol.

[39] Song Y, Luo Q, Long H, Hu Z, Que T, Zhang X A, Li Z, Wang G, Yi L, Liu Z, Fang W, Qi S (2014). Alpha-enolase as a potential cancer prognostic marker promotes cell growth, migration, and invasion in glioma. Mol Cancer.

[40] Vander H M G, Cantley L C, Thompson C B (2009). Understanding the Warburg Effect: The Metabolic Requirements of Cell Proliferation. Science.

[41] Yang J, Zhou M, Zhao R, Peng S, Luo Z, Li X, Cao L, Tang K, Ma J, Xiong W, Fan S, Schmitt D C, Tan M, Li X, Li G (2014). Identification of candidate biomarkers for the early detection of nasopharyngeal carcinoma by quantitative proteomic analysis. J Proteomics.

[42] Zhan P, Zhao S, Yan H, Yin C, Xiao Y, Wang Y, Ni R, Chen W, Wei G, Zhang P (2017). a-enolase promotes tumorigenesis and metastasis via regulating AMPK/mTOR pathway in colorectal cancer. Mol Carcinog.

[43] Thomas J D, Zhang Y, Wei Y, Cho J, Morris L E, Wang H, Zheng X (2014). Rab1A Is an mTORC1 Activator and a Colorectal Oncogene. Cancer Cell.

[44] Almohanna T, Al-Aadily I (2021). RNA Editing with CRISPR Cas13. Academia Letters.

